# Local IL-13 gene transfer prior to immune-complex arthritis inhibits chondrocyte death and matrix-metalloproteinase-mediated cartilage matrix degradation despite enhanced joint inflammation

**DOI:** 10.1186/ar1502

**Published:** 2005-01-26

**Authors:** Karin CAM Nabbe, Peter LEM van Lent, Astrid EM Holthuysen, Annet W Sloëtjes, Alisa E Koch, Timothy RDJ Radstake, Wim B van den Berg

**Affiliations:** 1 Department of Experimental Rheumatology and Advanced Therapeutics, University Medical Center Nijmegen, Nijmegen, The Netherlands; 2 University of Michigan Medical School, Ann Arbor, Michigan, USA; and Veterans Administration Ann Arbor, Ann Arbor, Michigan, USA

**Keywords:** cartilage destruction, experimental arthritis, interleukin-13, Fcγ receptors, MMPs

## Abstract

During immune-complex-mediated arthritis (ICA), severe cartilage destruction is mediated by Fcγ receptors (FcγRs) (mainly FcγRI), cytokines (e.g. IL-1), and enzymes (matrix metalloproteinases (MMPs)). IL-13, a T helper 2 (Th2) cytokine abundantly found in synovial fluid of patients with rheumatoid arthritis, has been shown to reduce joint inflammation and bone destruction during experimental arthritis. However, the effect on severe cartilage destruction has not been studied in detail. We have now investigated the role of IL-13 in chondrocyte death and MMP-mediated cartilage damage during ICA. IL-13 was locally overexpressed in knee joints after injection of an adenovirus encoding IL-13 (AxCAhIL-13), 1 day before the onset of arthritis; injection of AxCANI (an empty adenoviral construct) was used as a control. IL-13 significantly increased the amount of inflammatory cells in the synovial lining and the joint cavity, by 30% to 60% at day 3 after the onset of ICA. Despite the enhanced inflammatory response, chondrocyte death was diminished by two-thirds at days 3 and 7. The mRNA level of FcγRI, a receptor shown to be crucial in the induction of chondrocyte death, was significantly down-regulated in synovium. Furthermore, MMP-mediated cartilage damage, measured as neoepitope (VDIPEN) expression using immunolocalization, was halved. In contrast, mRNA levels of MMP-3, -9, -12, and -13 were significantly higher and IL-1 protein, which induces production of latent MMPs, was increased fivefold by IL-13. This study demonstrates that IL-13 overexpression during ICA diminished both chondrocyte death and MMP-mediated VDIPEN expression, even though joint inflammation was enhanced.

## Introduction

One of the main pathological features of rheumatoid arthritis is marked destruction of cartilage [1]. This destruction starts with reversible proteoglycan depletion, which is followed by irreversible cartilage degradation defined as chondrocyte death and breakdown of collagen type II, eventually leading to matrix erosion. The latter is mainly induced by matrix metalloproteinases (MMPs), which generate specific cleavage sites within matrix molecules [2,3]. MMPs are secreted in an inactive form by IL-1-stimulated chondrocytes, synovial macrophages, and fibroblasts [4-6]. Activation of MMPs is still poorly understood, but MMP activity is primarily found in experimental immune-complex (IC)-dependent arthritis models.

Immunoglobulin G (IgG)-containing ICs can activate macrophages upon recognition by Fcγ receptors (FcγRs). Three classes of murine FcγR can be distinguished: FcγRI, II, and III. Triggering FcγRI and III activates cellular responses, whereas FcγRII is an inhibitory receptor [7]. Previous studies have showed that activating FcγRI and III are crucial in induction of severe cartilage destruction, since chondrocyte death and MMP-mediated cartilage damage were absent in FcγR-deficient mice after induction of immune-complex-mediated arthritis (ICA) [8]. Furthermore, cartilage damage is aggravated by local overexpression of the proinflammatory T helper (Th)1 cytokine IFNγ [9]. This increase in cartilage destruction was observed only in IC-dependent arthritis models [9]. FcγRI was found to be crucial in the induction of chondrocyte death, whereas both FcγRI and III mediated MMP-mediated expression of VDIPEN [9].

Since the Th1 cytokine IFNγ worsens the arthritic response by up-regulation of the activating FcγRs, overexpression of a Th2 cytokine during arthritis might be protective, because of down-regulation of these receptors. In earlier studies, we found that adenoviral overexpression of IL-4 resulted in reduced MMP-mediated cartilage damage and chondrocyte death during ICA and arthritis induced by collagen type II [10,11]. IL-4 is regarded as a potent anti-inflammatory cytokine by direct inhibition of proinflammatory cytokines such as IFNγ, IL-1, and tumor necrosis factor α [12]. However, IL-4 protein and mRNA are hardly detected in synovial fluid and synovium of rheumatoid arthritis patients [13]. In contrast, IL-13 is expressed in rheumatoid arthritis synovial fluid and synovial fluid macrophages and resembles many functions of IL-4 [14,15]. Systemic overexpression of IL-13 in collagen-type-II-induced arthritis and local overexpression of IL-13 in rat adjuvant-induced arthritis reduced joint inflammation and bone destruction [16,17]. However, the effect of IL-13 on cartilage destruction was not investigated in detail in these studies and remains to be elucidated.

In the present study, we investigated whether IL-13 influences the development of chondrocyte death and MMP-mediated VDIPEN expression in ICA. Subsequently, regulation of FcγR, MMP, and IL-1 expression by IL-13 was studied, as these are important mediators in severe cartilage damage.

The present study demonstrates that overexpression of IL-13 in arthritic knee joints reduces chondrocyte death and MMP-mediated VDIPEN expression despite enhanced joint inflammation. Injection of an adenovirus encoding for IL-13 diminished chondrocyte death, which correlated with down-regulation of FcγRI expression in the synovium. Reduction of MMP-mediated VDIPEN expression was not reflected by MMP mRNA and IL-1 concentrations, as these were increased.

## Materials and methods

### Animals

C57Bl/6 male mice (10 to 12 weeks old) were purchased from Elevage-Janvier (Le Genest Saint Isle, France). Mice were fed a standard diet and tap water ad libitum. Ethical approval was obtained from the research ethics committee of the Central Animal Facility in Nijmegen.

### Local gene transfer of IL-13

The recombinant adenovirus encoding human IL-13 (AxCAhIL-13) was generated as described before [17-19] and an empty adenoviral construct (AxCANI) was used as control virus. AxCAhIL-13 or AxCANI (1.10^7 ^plaque-forming units) was injected intra-articularly in naive knee joints. Patellae with adjacent synovium were dissected in a standardized manner [20] and synovial biopsies were taken with a biopsy punch (diameter of 3 mm). Total RNA was extracted in 1 ml TRIzol reagent and used for quantitative PCR as described below. AxCAhIL-13 or AxCANI was injected intra-articularly 1 day before the induction of arthritis.

### Induction of immune-complex-mediated arthritis

Rabbit polyclonal antibodies directed against lysozyme were injected intravenously into mice. ICA was then passively induced by injecting 3 μg lysozyme coupled to poly-L-lysine in 6 μl pyrogen-free saline into the knee joints.

### Histology of arthritic knee joints

Total knee joints were dissected at days 3 and 7 after the onset of arthritis. Joints were decalcified, dehydrated, and embedded in paraffin. Tissue sections (7 μm) were stained with hematoxylin and eosin.

Histopathological changes were scored in two ways. Inflammation was graded on a scale from 0 (no inflammation) to 3 (severely inflamed joint) as influx of inflammatory cells in synovium and joint cavity. Chondrocyte death was scored as the amount of empty lacunae expressed as a percentage of the total number of cells within the cartilage layers.

### Immunohistochemical detection of macrophages and polymorphonuclear neutrophils (PMNs)

Macrophages were detected using a specific antibody against F4/80, a murine macrophage membrane antigen [21]. PMNs were visualized using NIMPR14, a specific rat anti-mouse monoclonal antibody [22]. Primary antibodies were detected using rabbit anti-rat IgG and avidin–horseradish peroxidase conjugate. Finally, sections were counterstained with hematoxylin. Macrophage and PMN subsets were quantitatively measured using an image analysis system. The inflammatory cell mass was selected by hand and the amount of positive features present in this area was displayed using a computer imaging system. Three sections of each knee joint were measured and the mean was calculated. We report the amount of positive features per 100,000 μm^2 ^inflammatory cell mass in the synovium.

### Immunohistochemical VDIPEN staining

Sections were digested with proteinase-free chondroitinase ABC (0.25 units/ml in 0.1 M Tris/HCl, pH 8.0; Sigma, Zwijndrecht, The Netherlands) to remove the side chains of proteoglycans followed by incubation with affinity-purified rabbit anti-VDIPEN IgG [23]. The primary antibody was detected using biotinylated goat anti-rabbit IgG, and avidin–streptravidin–peroxidase (Elite kit; Vector, Burlingame, CA, USA). Counterstaining was done with orange G (2%). Areas of immunostaining were expressed as a percentage of the total cartilage surface.

### Quantitative detection of FcγR and MMP mRNA using RT-PCR

Specific mRNA levels for FcγRI, II, and III and MMP-3, -9, -12, -13, and -14 were detected using the ABI/PRISM 7000 Sequence Detection System (ABI/PE; Foster City, CA, USA). Briefly, 1 μg of synovial RNA was used for RT-PCR. mRNA was reverse transcribed to cDNA using oligodT primers. cDNA (1/100) was used in one PCR amplification. PCR was performed in SYBR Green Master Mix using the following amplification protocol: 2 min at 50°C followed by 40 cycles of 15 s at 95°C and 1 min at 60°C with data collection in the last 30 s. Message for murine FcγRI, II, and III and MMP-3, -9, -12, -13, and -14 was amplified using the primers listed in Table 1 (Biolegio, Malden, The Netherlands) at a final concentration of 300 nmol/l. Relative quantification of the PCR signals was performed by comparing the cycle threshold value (Ct) of the FcγR and MMP genes in the different samples after correction of the GAPDH content for each individual sample.

### Determination of cytokine and chemokine concentrations

To determine concentrations of IL-13, IL-1β, KC (a mouse homologue for human growth-related protein), and macrophage inflammatory protein 1α in patella washouts, synovial specimens were isolated in a standard manner [20] and incubated in 200 μl RPMI 1640 medium (GIBCO BRL, Breda, The Netherlands) for 1 hour at room temperature. Cytokine and chemokine concentrations were determined using the BioPlex^® ^system from BioRad (Hercules, CA, USA) for the Luminex^® ^multi-analyte system and expressed as pg/ml.

### Statistical analysis

Differences between experimental groups were tested for significance using the Mann–Whitney *U *test. *P *values <0.05 were considered statistically significant.

## Results

### Local IL-13 expression in naive knee joints using adenoviral gene transfer

**Figure 1:**
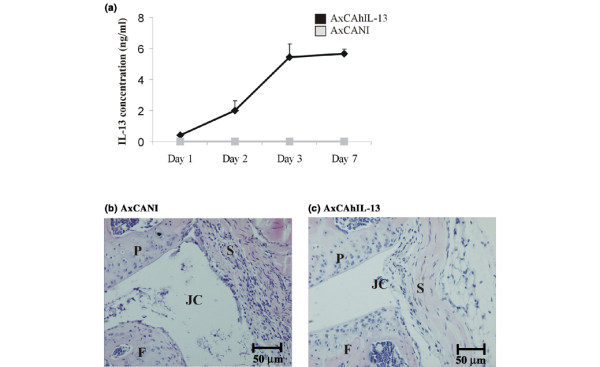
Adenoviral-vector-mediated IL-13 expression in knee joints of C57Bl/6 mice. **(a) **Naive knee joints and **(b) **total knee joint sections 24 hours after injection of AxCANI (adenovirus encoding no gene) or of **(c) **AxCAhIL-13 (adenovirus encoding interleukin-13). Injection of AxCAhIL-13 resulted in 0.4 ng/ml IL-13 at day 1, which increased to 5.5 ng/ml by day 7 (a). Injection of AxCANI resulted in a mild thickening of the synovial lining (S) and some invading inflammatory cells in the joint cavity (JC) (b), whereas no inflammation was observed after AxCAhIL-13 injection (c). Plotted values are means ± SEM of data from 5 mice. *P < 0.05. Original magnification 200×. F, femur; P, patella.

The expression of IL-13 was determined in synovial washouts at days 1, 2, 3, and 7 after injection of the AxCAhIL-13 virus. IL-13 reached a concentration of 0.4 ng/ml after 24 hours. Values increased to 2 ng/ml at day 2 and remained high up to 7 days after injection (Fig. 1a). IL-13 was not detected after injection of AxCANI.

We next investigated whether injection of the adenoviral IL-13 construct causes joint inflammation by itself. Using histology, we found that IL-13 overexpression in naive knee joints did not recruit inflammatory cells at day 1, 2, 3, or 7 (Fig. 1c). Injection of AxCANI resulted in minor cell influx in the synovial lining and joint cavity (Fig. 1b), which was not detectable from day 2 onwards.

### IL-13 overexpression during ICA enhances joint inflammation and alters the composition of the cell mass

To investigate whether IL-13 overexpression ameliorated the arthritic response, we injected AxCAhIL-13 1 day before ICA induction. Joint inflammation was studied 3 and 7 days after arthritis onset.

**Figure 2:**
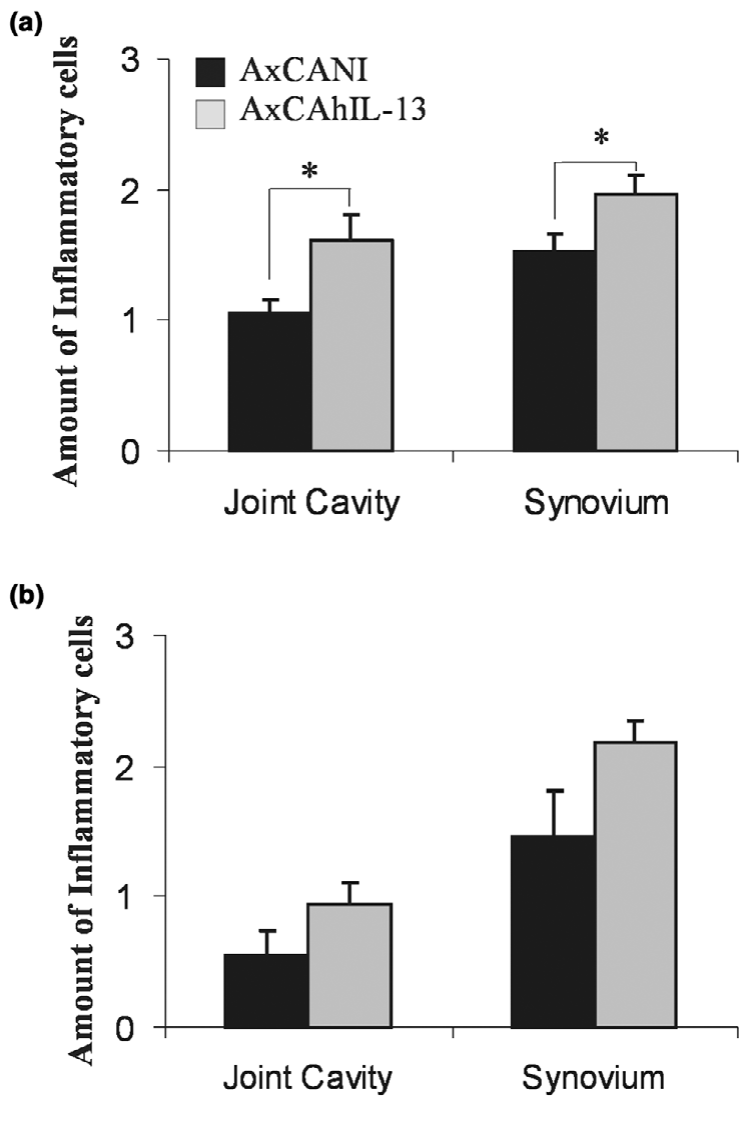
Joint inflammation in arthritic knee joints of C57Bl/6 mice injected with AxCANI (adenovirus encoding no gene) or AxCAhIL-13 (adenovirus encoding interleukin-13). At **(a) **day 3 and **(b) **day 7 after the onset of immune-complex-mediated arthritis. The inflammatory cell mass was significantly enhanced by IL-13 in both the joint cavity and the synovium 3 days after arthritis induction. Bars show the means ± SEM for 10 mice. Significance was evaluated using the Mann–Whitney *U *test. *P < 0.05.

IL-13 overexpression significantly increased the inflammatory cell mass in joint cavity and synovium, by 60% and 30%, respectively, 3 days after arthritis induction (Fig. 2a). After 7 days, joint inflammation seemed to normalize in the IL-13 group (Fig. 2b).

To further investigate inflammatory cell types attracted by IL-13, PMNs and macrophages were detected using specific NIMPR14 and F4/80 antibodies respectively using immunolocalization.

**Figure 3:**
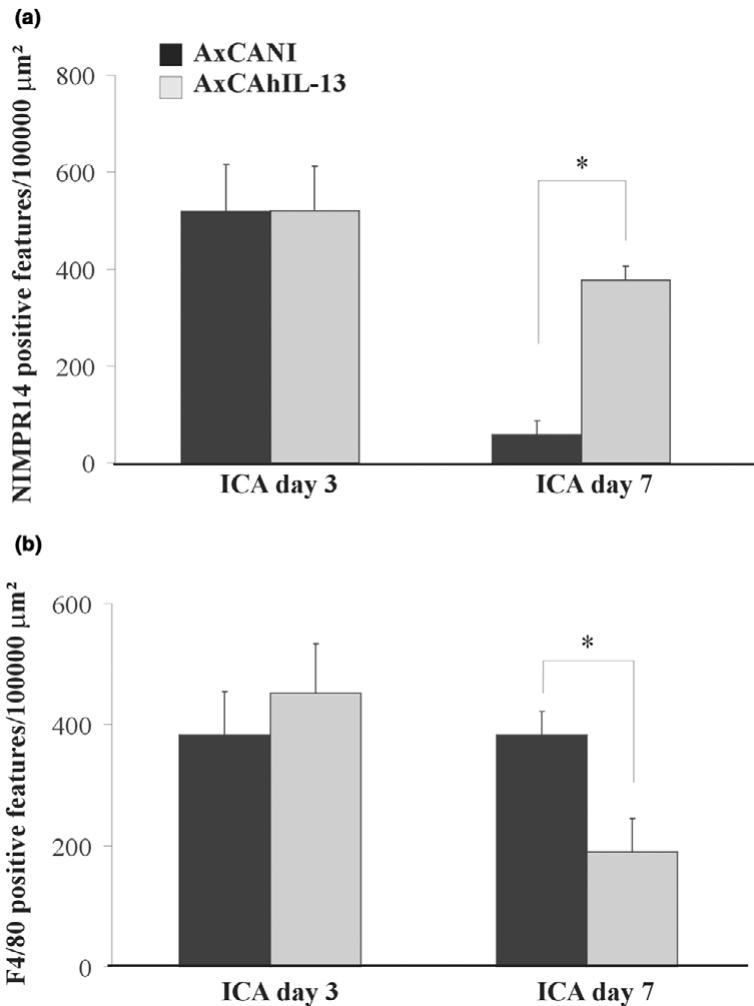
Immunohistochemical detection of inflammatory cells in knee joints of mice with immune-complex-mediated arthritis (ICA). **(a) **Polymorphonuclear neutrophils and **(b) **macrophages in synovium 3 and 7 days after injection of AxCANI (adenovirus encoding no gene) or AxCAhIL-13 (adenovirus encoding interleukin-13). Polymorphonuclear neutrophils were detected using the specific rat anti-mouse monoclonal antibody NIMPR14, and macrophages were detected using an antibody against the membrane marker F4/80. At day 7, the amount of NIMPR14-positive features was significantly higher in the synovium of AxCAhIL-13-injected arthritic knee joints, while the amount of F4/80-positive features was significantly lower. The bars represent means ± SEM for 10 mice. Data were evaluated using the Mann–Whitney *U *test. *P < 0.05.

At day 3, the amount of PMNs and macrophages was not markedly altered by IL-13 (Fig. 3a and 3B). At day 7, however, the amount of PMNs in the synovial lining was 10 times higher (Fig. 3a), whereas the amount of macrophages in the IL-13 group was half that in the mice without IL-13 (Fig. 3b).

### KC concentration in synovial washouts is augmented by IL-13

A possible mechanism by which IL-13 can increase joint inflammation in the presence of ICs is elevation of chemokine production. To investigate this, synovial washouts were done on days 3 and 7, and the chemokines KC (chemotactic for neutrophils) and macrophage inflammatory protein1α (chemotactic for macrophages) were measured. Local IL-13 overexpression increased KC concentrations 4- and 18-fold, respectively, at days 3 and 7 after arthritis induction, which correlates with the high amount of PMNs (Table 2). Macrophage inflammatory protein1α concentrations at day 3 were comparable between the control and IL-13 groups. At day 7, macrophage inflammatory protein1α expression was slightly increased by IL-13 (Table 2).

### IL-13 strongly inhibits chondrocyte death during ICA: down-regulation of FcγRI

Because IL-13 enhanced the inflammatory response, we next investigated the effect of IL-13 overexpression on cartilage destruction. A characteristic feature of irreversible cartilage damage is chondrocyte death; this was scored as the percentage of empty lacunae relative to the total amount of chondrocytes present in various cartilage layers in the knee joint.

**Figure 4:**
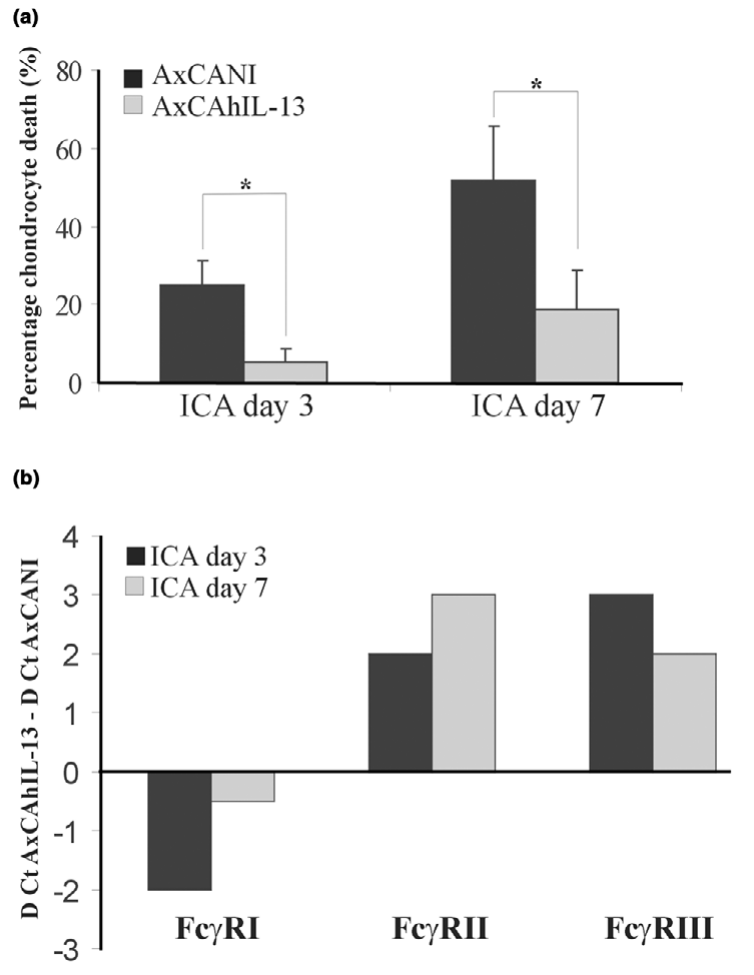
Chondrocyte death in the knee joints of mice with immune-complex-mediated arthritis (ICA). **(a) **At day 3 and 7 in arthritic knee joints injected with injected with AxCANI (adenovirus encoding no gene) or AxCAhIL-13 (adenovirus encoding interleukin-13) and **(b) **expression profiles of Fcγ receptor I (FcγRI), II, and III mRNA levels induced by IL-13 in synovium. IL-13 significantly decreased chondrocyte death, both at day 3 and at day 7 (a). Cycle threshold (Ct) values of FcγRI, II, and III in arthritic knee joints injected with AxCANI were subtracted from the Ct values for FcγRs after injection of AxCAhIL-13. Ct values were corrected for glyceraldehyde-3-phosphate dehydrogenase content for each individual sample. (b) FcγRI mRNA level was down-regulated by IL-13, whereas an up-regulation was observed for both FcγRII and III. Bars represent means ± SEM for 10 mice. Mann–Whitney *U *test. *P < 0.05. D, Δ.

Three days after ICA induction, chondrocyte death, expressed as the mean for six cartilage layers in the knee joint, was very low in the IL-13 group (5%) and significantly less than in the control arthritic knee joints, which showed 25% chondrocyte death (Fig. 4a). At day 7, chondrocyte death was even more significantly reduced (65%) in comparison with the control group (Fig. 4a).

In a previous study, we found that FcγRI is the dominant receptor mediating chondrocyte death during ICA [9]. We speculated that the decreased chondrocyte death might be caused by down-regulation of FcγRI by IL-13. For that reason, we determined the effect of IL-13 on mRNA levels of all three classes of FcγRs in synovium. Cycle values of FcγRI, II, and III in synovium of arthritic knee joints injected with AxCANI were subtracted from cycle values of FcγRs after AxCAhIL-13 injection. Interestingly, FcγRI mRNA level was decreased by IL-13 at day 3 after ICA induction (ΔCt = 2), and was still slightly down-regulated at day 7 (ΔCt = 0.5). In contrast, FcγRII and FcγRIII were up-regulated by IL-13, at both days 3 and 7 after ICA induction (Fig. 4b).

### IL-13 increases IL-1 production and MMP mRNA levels in the arthritic knee joint

Cartilage matrix degradation is largely mediated by MMPs. Production of latent MMPs is mainly regulated by IL-1 and this cytokine has been shown to be crucial in the generation of MMP-mediated neoepitopes [23]. The production of IL-1 was determined in synovial washouts of arthritic knee joints at both days 3 and 7. At day 3, IL-1 concentration was between 450 and 500 pg/ml in both the control and the IL-13 group. However, at day 7, the IL-1 concentration was reduced in the control group but remained high in the IL-13 group (control 54 pg/ml vs IL-13 255 pg/ml).

This sustained IL-1 production at day 7 may result in high concentrations of MMPs in synovium. Levels of MMP-3, -9, -12, -13, and -14 mRNA were detected by quantitative PCR. MMP-12 mRNA levels were increased 10-fold and 8-fold by IL-13 at days 3 and 7, respectively, after the onset of ICA. At day 7, mRNA levels of MMP-3, -9, and -13 were also significantly increased in the IL-13 group (Table 3).

### MMP-mediated VDIPEN expression is reduced by IL-13 overexpression

Increased IL-1 and MMP concentrations may induce enhanced MMP-mediated proteoglycan degradation and this was further investigated by detection of VDIPEN neoepitope expression in the cartilage.

**Figure 5:**
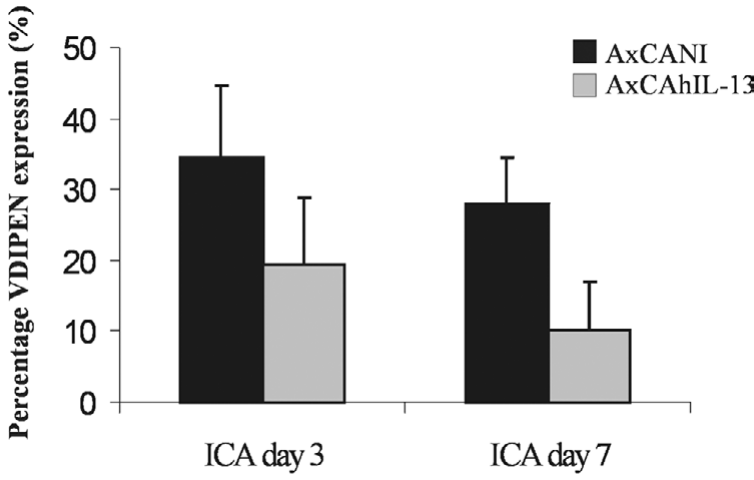
Matrix-metalloproteinase-mediated aggrecan damage in knee joints of mice with immune-complex-mediated arthritis. VDIPEN expression at day 3 and 7 after the induction of immune-complex-mediated arthritis in knee joints injected with AxCANI or AxCAhIL-13. Note that VDIPEN expression was reduced by IL-13 both at day 3 and day 7. Values represent the mean ± SEM for 10 mice. *P < 0.05, Mann–Whitney *U *test. AxCAhIL-13 = adenovirus encoding interleukin-13; AxCANI = adenovirus encoding no gene.

In the control group, 35% of the cartilage surface expressed VDIPEN neoepitopes after 3 days (Fig. 5). Injection with AxCAhIL13 reduced VDIPEN expression by 43%, as only 20% VDIPEN expression was found in the IL-13 group. The inhibitory effect of IL-13 was still present at day 7 after arthritis induction, as only 10% VDIPEN expression was found in the IL-13 group compared to 25% in the control group (Fig. 5).

## Discussion

In the present study, we have shown that local gene transfer of IL-13 reduced severe cartilage destruction defined as chondrocyte death and MMP-mediated aggrecan damage during ICA.

Local IL-13 overexpression during IC-dependent arthritis enhanced joint inflammation. To exclude the possibility that IL-13 itself induces influx of inflammatory cells, as is found when IL-13 is overexpressed in the lung [24,25], AxCAhIL-13 was injected in naive knee joints. We observed that IL-13 overexpression in the knee joint did not recruit inflammatory cells. This observation indicates that overexpression of IL-13 induces elevated joint inflammation in combination with IC triggering. In our IC-dependent arthritis model, we showed that joint inflammation is determined by activating FcγRIII [26]. In the present study, we find that IL-13 increased expression of FcγRIII within the synovium, which is not in line with the study showing that IL-13 decreases FcγRIII expression on human monocytes [27].

However, regulation of FcγR expression on mouse macrophages by IL-13 has not been described. IL-13 has high similarity with IL-4, which can increase FcγRIII expression on murine mast cells [28]. Binding of IC to FcγRIII on macrophage lining cells leads to activation, resulting in elevated influx of inflammatory cells. We further found that overexpression of IL-13 in arthritic knee joints particularly increased the amount of PMNs. This is in line with earlier studies in which it was shown that stimulation of FcγRIII induces release of PMN attracting chemokines as IL-8, resulting in neutrophil accumulation [29-31].

The proinflammatory action of IL-13 found in the present study seems to be dependent on costimulation with ICs to trigger arthritis onset, since local overexpression of IL-13 during T-cell-mediated rat adjuvant-induced arthritis diminishes joint inflammation [17]. In the latter model, ICs do not play a role. Whether IL-13 decreases or enhances joint inflammation may also be dependent on systemic or local overexpression. Systemic overexpression of IL-13 during collagen-type-II-induced arthritis, in which FcγRIII is also required for arthritis development [32], decreased joint inflammation [16]. An explanation may be that systemic overexpression of IL-13 hampers the development of the immune response by induction of isotype switching to the nonarthritogenic IgG4 and IgE [33,34], thereby ameliorating the arthritic response. Induction of immunity is hardly affected by local overexpression, as was shown when injection of AdIL-4 (adenovirus expressing IL-4) in knee joints during arthritis induced by collagen type II markedly increased the amount of inflammatory cells [11].

Cartilage destruction during ICA is mostly related to joint inflammation. Despite the enhanced influx of inflammatory cells, however, a significant reduction of chondrocyte death was induced by IL-13. Chondrocyte death may be the result of increased production of oxygen radicals, as reactive oxygen species can mediate apoptosis [35]. In a previous study, we showed that there is a prominent role for FcγRI mediating chondrocyte death during ICA. In FcγRI-deficient mice, chondrocyte death was almost absent. When the Th1 cytokine IFNγ was overexpressed, a significant increase in chondrocyte death was observed, which was dependent on FcγRI [9]. Stimulation of FcγRI leads to production of oxygen radicals via NADPH-oxidase [36]. In the present study, we find that in knee joints injected with AxCAhIL-13, FcγRI expression remained low, whereas in knee joints injected with control virus, FcγRI expression level was enhanced in the synovium. The decrease in chondrocyte death might be due to a reduced FcγRI concentration. Moreover, it has been shown that IL-13 itself down-regulates production of oxygen radicals by inflammatory cells, since IL-13 can inhibit protein-kinase-C-triggered respiratory burst in monocytes [37]. The inhibiting effect of IL-13 on oxygen radical production seemed to be monocyte-dependent, as no reduction was found in PMNs [38].

In addition, IL-13 also reduced MMP-mediated VDIPEN neoepitope expression. It has been reported that IL-13 diminishes the breakdown of collagen and proteoglycans from bovine cartilage, by regulation of MMP expression [39]. Several mechanisms may inhibit MMP-mediated cartilage destruction, as regulation of MMPs occurs at three different levels: MMP synthesis, activation of latent enzyme, and MMP inhibition. IL-1 is a prominent cytokine controlling the production of latent MMPs [40], and diminished production of IL-1 might reduce MMP-mediated cartilage damage. We found, however, that IL-13 overexpression in arthritic knee joints strongly increased IL-1β concentrations. IL-13 is described as an anti-inflammatory cytokine, which in general reduces IL-1β production [14,27,41]. However, the effect of IL-13 on IL-1 production by IC-stimulated macrophages has not been described to date. In addition to macrophages, fibroblasts and PMNs are also present in the knee joint at day 7 after the onset of arthritis.

The sustained production of IL-1 by IL-13 may indeed stimulate MMP production, as reflected by enhanced MMP-3, -9, -12, and -13 mRNA levels 7 days after ICA induction in AxCAhIL-13-injected arthritic knee joints. MMP-12 mRNA level was already increased at day 3 after the onset of arthritis. It has been shown that MMP-12 expression is IL-13-dependent and that MMP-12 is a critical downstream mediator and regulator of IL-13-induced responses [42,43]. Furthermore, IL-13 induction of MMP-2, -9, and -13 is at least partly mediated by MMP-12 [43], indicating that MMP-12 may be a crucial enzyme inducing MMP-mediated cartilage damage.

Furthermore, IL-13 might interfere at the level of activation of MMPs. MMPs are secreted in a latent form and activation occurs after cleavage of a propeptide. Factors that activate latent MMPs are still unknown. However, MMP-mediated VDIPEN expression is mainly found in IC-dependent arthritis models, in which FcγRs are of utmost importance. Down-regulation of the activating FcγRs might reduce VDIPEN expression. Indeed, we found that IL-13 strongly diminished FcγRI expression in synovium. Another mechanism involved in activation of MMPs is production of oxygen radicals. As mentioned above, stimulation of FcγRI results in assembly of the NADPH-oxidase complex, which produces oxygen radicals [36]. Additionally, oxygen metabolites can be converted into H_2_O_2_, which can activate latent proMMPs [44,45]. Taken together, decreased FcγRI expression reduces the production of oxygen radicals, which apart from chondrocyte protection may also result in diminished MMP-mediated VDIPEN expression.

## Conclusion

The present study shows that IL-13 is a potent cytokine that protects the cartilage matrix against degradation during ICA. In addition, these results indicate that regulation of the expression of FcγR, particularly FcγRI, might be involved in this process. Therefore, modulation of FcγRI by Th2 cytokines seems to be a promising therapeutic tool diminishing cartilage damage in rheumatoid arthritis.

## Abbreviations

AxCAhIL-13 = adenovirus encoding interleukin-13; AxCANI = adenovirus encoding no gene; Ct = cycle threshold; FcγR = Fcγ receptor; IC = immune complex; ICA = immune-complex-mediated arthritis; IFNγ = interferon γ; IgG = immunoglobulin G; IL = interleukin; KC = mouse homologue for human IL-8; MMP = matrix metalloproteinase; NADPH = reduced nicotinamide adenine dinucleotide phosphate; PMN = polymorphonuclear neutrophil; RT-PCR = reverse transcriptase polymerase chain reaction; Th, T helper.

## Competing interests

The author(s) declare that they have no competing interests.

## Authors' contributions

KN designed the experimental design of the study, carried out the experiments, and drafted the manuscript. PL participated in the experimental design of the study and preparation of the manuscript. AH participated in the animal studies. AS participated in isolation of mRNA and performing PCRs. AK provided the adenoviruses and participated in the preparation of the manuscript. TR participated in the preparation of the manuscript. WB participated in the design of the study and preparation of the manuscript. All authors read and approved the final manuscript.

## Figures and Tables

**Table 1:** Primers for detection of murine FcγRI, II, and III mRNA

Gene	Primer	Primer sequence
GAPDH	Up	5'-GGC-AAA-TTC-AAC-GGC-ACA-3'
	Low	5'-GTT-AGT-GGG-GTC-TCG-CTC-CTG-3'
		
FcγRI	Up	5'-ACA-CAA-TGG-TTT-ATC-AAC-GGA-ACA-3'
	Low	5'-TGG-CCT-CTG-GGA-TGC-TAT-AAC-T-3'
		
FcγRII	Up	5'-GAC-AGC-CGT-GCT-AAA-TCT-TGC-T-3'
	Low	5'-GTG-TCA-CCG-TGT-CTT-CCT-TGA-G-3'
		
FcγRIII	Up	5'-GAC-AGG-CAG-AGT-GCA-GCT-CTT-3'
	Low	5'-TGT-CTT-CCT-TGA-GCA-CCT-GGA-T-3'
		
MMP-3	Up	5'-TGG-AGC-TGA-TGC-ATA-AGC-CC-3'
	Low	5'-TGA-AGC-CAC-CAA-CAT-CAG-GA-3'
		
MMP-9	Up	5'-GGA-ACT-CAC-ACG-ACA-TCT-TCC-A-3'
	Low	5'-GAA-ACT-CAC-ACG-CCA-GAA-GAA-TTT-3'
		
MMP-12	Up	5'-GGA-CAT-GAA-GCG-TGA-GGA-TGT-3'
	Low	5'-GAA-GTC-TCC-GTG-AGC-TCC-AAA-T-3'
		
MMP-13	Up	5'-ACC-TTG-TGT-TTG-CAG-AGC-ACT-AAC-TT-3'
	Low	5'-CTT-CAG-GAT-TCC-CGC-AAG-AGT-3'
		
MMP-14	Up	5'-AAG-GCT-GAT-TTG-GCA-ACC-AT-3'
	Low	5'-GTC-CCA-AAC-TTA-TCC-GGA-ACA-C-3'

Primer sequences used for RT-PCR on synovium. FcγR, Fcγ receptor; GAPDH, glyceraldehyde-3-phosphate dehydrogenase; MMP, matrix metalloproteinase.

**Table 2:** Effect of IL-13 on chemokine concentrations (pg/ml) in arthritic joints in mice

Group	KC	MIP1α
ICA, day 3		
AxCANI	56 ± 8	303 ± 6.8
AxCAhIL-13	196 ± 31*	344 ± 96
ICA, day 7		
AxCANI	10 ± 6	157 ± 25
AxCAhIL-13	184 ± 26*	268 ± 98

Concentrations of KC and MIP1α were detected in synovial washouts of arthritic knee joints 3 and 7 days after arthritis induction. KC concentrations were significantly higher in arthritic knee joints injected with AxCAhIL-13 both at day 3 and 7. *P < 0.05 in comparison with AxCANI. AxCAhIL-13, adenovirus encoding interleukin-13; AxCANI, adenovirus encoding no gene; ICA, immune-complex-mediated arthritis; KC, mouse homologue for human IL-8; MIP 1α, macrophage inflammatory protein 1α.

**Table 3:** Effect of IL-13 on MMP mRNA levels in synovium of mice with ICA

	ICA day 3		ICA day 7	
	AxCANI	AxCAhIL-13	AxCANI	AxCAhIL-13

MMP-3	5.7 ± 0.3	7.1 ± 0.8	4.2 ± 0.5	6.1 ± 0.2*
MMP-9	5.1 ± 0.2	4.8 ± 0.3	0.2 ± 0.6	3.9 ± 0.5*
MMP-12	0.6 ± 0.4	5.8 ± 1.1*	0.9 ± 1	8.1 ± 0.9*
MMP-13	3.2 ± 0.2	2.7 ± 0.3	4.3 ± 0.3	6.4 ± 0.3*
MMP-14	3.7 ± 0.4	4.9 ± 0.8	3.7 ± 1	3.7 ± 0.6

Expression profile of MMP-3, -9, -12, -13, and -14 mRNA levels after injection of AxCANI or AxCAhIL-13 in synovial biopsies isolated at day 3 and day 7 after arthritis onset. The Ct values for MMP genes in naive knee joints were subtracted from the Ct values for MMPs at day 3 and 7 after arthritis onset. Ct values were corrected for GAPDH content for each individual sample. Note that MMP-3, -9, -12, and -13 mRNA levels were significantly increased at day 7 by IL-13, and the MMP-12 mRNA level was already elevated at day 3. Values represent means ± SEM for 5 mice. *P < 0.05, Mann–Whitney *U *test. AxCAhIL-13 = adenovirus encoding interleukin-13; AxCANI = adenovirus encoding no gene; Ct = cycle threshold; GAPDH = glyceraldehyde-3-phosphate dehydrogenase; ICA = immune-complex-mediated arthritis; MMP = matrix metalloproteinase.
